# Application of Surface Electromyography (sEMG) in the Analysis of Upper Limb Muscle Activity in Women Aged 50+ During Torqway Riding

**DOI:** 10.3390/s25144280

**Published:** 2025-07-09

**Authors:** Sylwia Agata Bęczkowska, Iwona Grabarek, Zuzanna Zysk

**Affiliations:** Faculty of Transport, Warsaw University of Technology, 00-662 Warsaw, Poland; iwona.grabarek@pw.edu.pl (I.G.); zuzanna.zysk@pw.edu.pl (Z.Z.)

**Keywords:** active transport, sensors, sEMG analysis, muscle synergy, sustainable mobility, physical activity, rehabilitation

## Abstract

The aim of this study was to analyze the activation of selected upper limb muscles. For the purposes of this article, we present results concerning the following muscles: triceps brachii, anterior and posterior deltoid, and trapezius in women aged 50 and above during simulated riding of the Torqway device, using surface electromyography (sEMG). The primary objective was to compare muscle activity across two movement phases: active and passive. Accordingly, the following research hypotheses were formulated: muscle activity (measured by RMS values) will be significantly higher during the active phase compared to the passive phase, and MPF (mean power frequency) values will decrease over time, indicating the onset of muscle fatigue. Additionally, the potential of surface electromyography was assessed as a diagnostic tool for evaluating ergonomics and muscle effort in the context of designing personalized mobility devices for older adults. As the study of the Torqway device represents a pioneering research effort, this publication makes a significant contribution to the biomechanical analysis of new forms of active mobility supported by wearable sensor technologies.

## 1. Introduction

The growing demand for sustainable and health-promoting transport solutions has led to an increased interest in personal mobility devices that support physical activity, particularly among aging populations.

The endeavor to change society’s mindset regarding transportation choices is a long-term process. The first step is a shift in attitude towards public transportation, followed by the utilization of individual means of transportation, such as bicycles or scooters. To enrich the range of individual transportation options, designers propose new solutions with an ecological emphasis, but primarily solutions that contribute to enhancing physical fitness, especially among individuals aged 60+ [1,2,3,4].

Interest and willingness to use such vehicles must be preceded by credible research demonstrating their benefits, as well as their impact on users’ physical condition, particularly on the muscular system [5,6].

One of the currently used techniques for assessing muscle activity is surface electromyography (sEMG), which focuses on measuring and analyzing myoelectric signals using surface electrodes [7,8,9]. Surface electromyography (sEMG) enables the assessment of muscle loading and fatigue based on the analysis of electrical activity of stimulated muscles. Muscle fatigue is typically defined as a reduction in the muscle’s ability to generate force. More precisely, it is a reversible decline in the muscle’s capacity to produce force over time due to sustained or repetitive activation. In surface EMG analysis, fatigue is often characterized by an increase in signal amplitude (RMS) and a decrease in mean power frequency (MPF), reflecting altered motor unit recruitment and conduction velocity [10,11]. From an sEMG perspective, fatigue is often associated with an increase in RMS values and a decrease in MPF, although this interpretation requires caution.

The sEMG signal is influenced by both internal (personal) and external factors. Internal factors include the conduction speed of muscle fiber action potentials, switching and synchronization of individual motor units, as well as anatomical factors such as subcutaneous layer thickness (fat tissue content) and the proportion of muscle fibers of a particular type. External factors are associated with the applied external force, duration of load maintenance, type of measuring electrodes used, and electrical resistance between the electrode and the skin. Additionally, the sEMG signal depends on geometric factors affecting muscle length changes (in the case of analyzed studies resulting from changes in body position and the technique of riding the examined device). Among the factors influencing the sEMG signal, the physical activity of the subjects should also be highlighted. The signals received result from physiological changes in the state of muscle fiber cell membranes [1,10,11,12]. In contrast to classical neurological sEMG, where the artificial response of muscles to external electrical stimulation is examined under static conditions, kinesiological sEMG allows for the examination of neuromuscular activity during tasks related to posture, functional movements, work, and treatment/training [13,14,15,16]. In addition to basic physiological and biomechanical research, kinesiological sEMG serves as an assessment tool in scientific studies within the scope of physiotherapy/rehabilitation, sports, training, ergonomics, as well as human body interaction with industrial products and work conditions [17,18,19].

This versatile tool has become an indispensable element in the fields of medicine, rehabilitation, sports, and ergonomic research. Consequently, research on human muscle activity during individual transportation was conducted at the Faculty of Transport of the Warsaw University of Technology using the aforementioned method. The research was commissioned by TORQWAY, a company implementing the construction of a hybrid personal mobility device for standing movement called “Torqway” as part of the European Union’s Horizon 2020 research and innovation program, project number: 778154 (SMEI H2020) [20]. Torqway is an invention patented in Poland in 2011. It was primarily created for sports and recreational purposes. Promoting sustainable mobility involves not only reducing dependence on fossil fuels but also encouraging transportation solutions that enhance users’ health and reduce the environmental footprint. Active transport modes, such as walking, cycling, and human-powered vehicles, play a vital role in achieving urban sustainability goals. Devices like the Torqway bridge the gap between mobility support and physical activity, making them particularly valuable for older populations.

Torqway is suitable for use wherever pedestrians and cyclists move: on bike paths and promenades, on forest trails and in residential areas, in park avenues and city squares, as well as on scenic routes outside the city.

**Figure 1:**
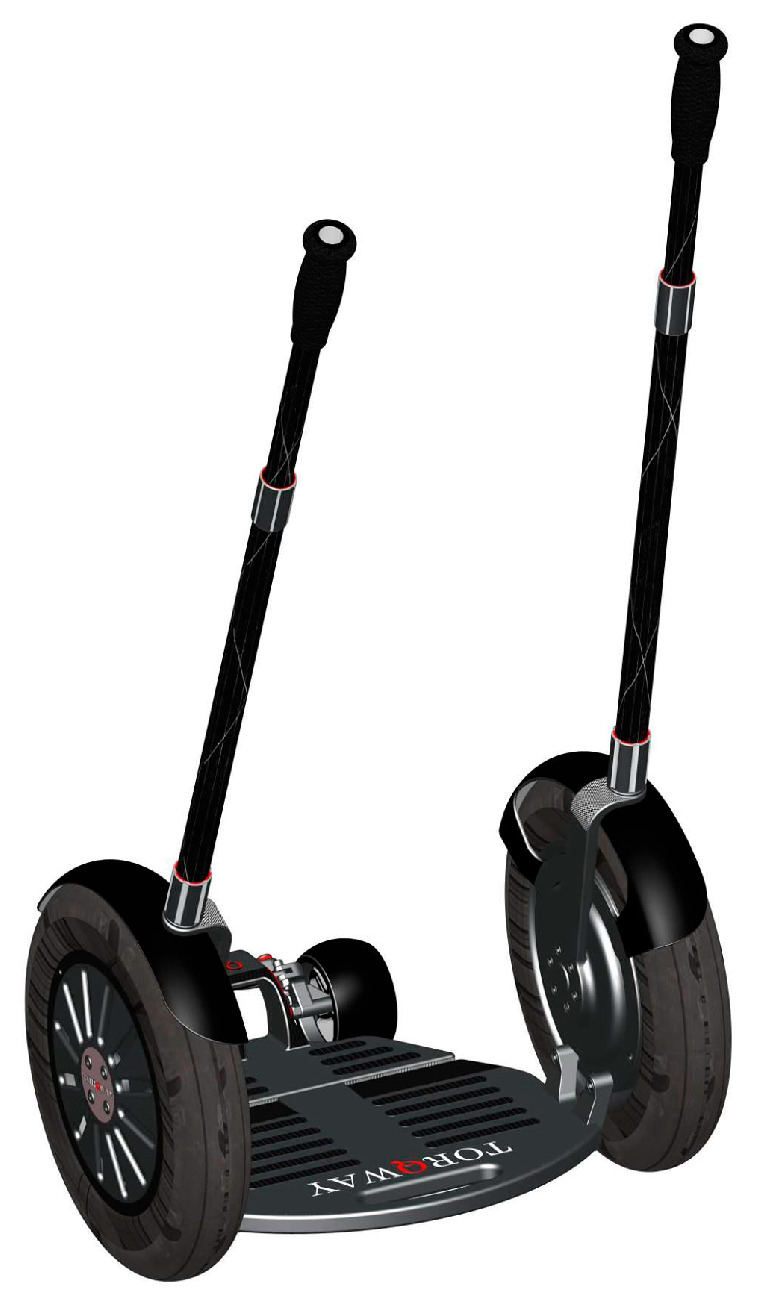
Torqway personal mobility device. Source: Adapted from https://antyweb.pl/polacy-z-torunia-stworzyli-torqway-to-bedzie-wiekszy-hit-niz-segway (accessed on 20 May 2025).

What distinguishes it from scooters, Segways, or electric scooters is the fact that its use engages the muscles of the upper and lower limbs as well as the torso. It is a hand-powered vehicle that allows for movement in a standing position, similar to a Segway. Moving the levers forward and backward while riding generates a unidirectional rotational movement of the wheel axle, causing the vehicle to move forward. Activity on the Torqway combines elements of Nordic walking and using an orbital-type device. Since the device is easy to operate, it can be used by children, adults, and seniors. The broad range of potential users significantly distinguishes Torqway from other individual means of transportation (Figure 1). Currently, a hybrid version is also available, which can be used by physically weak individuals.

The length and frequency of lever movement depend on the user’s preference. The resistance of the lever is moderated. The research included an evaluation of the user’s body movement kinematics, the results of which were published in [21], and an analysis of muscle tension, presented in this article. The aim of this study was to evaluate muscle activation patterns and potential fatigue in selected upper limb muscles—namely the triceps brachii, anterior and posterior deltoid, and trapezius—in women aged 50 and above during simulated Torqway riding using surface electromyography (sEMG). The analysis focused on two movement phases: active and passive. Accordingly, the following research hypotheses were formulated: muscle activity (measured by RMS values) will be significantly higher during the active phase compared to the passive phase, and MPF (mean power frequency) values will decrease over time, indicating the onset of muscle fatigue. Additionally, the potential of surface electromyography was assessed as a diagnostic tool for evaluating ergonomics and muscle effort in the context of designing personalized mobility devices for older adults. As the study of the Torqway device represents a pioneering research effort, this publication makes a significant contribution to the biomechanical analysis of new forms of active mobility supported by wearable sensor technologies.

The present research is situated within the emerging paradigm of sensor-integrated biomechanics and dynamic physiology, where the use of wearable technologies, such as surface electromyography (sEMG), plays a transformative role in evaluating human performance. By bridging engineering and biological systems, wearable sensors allow for a high-resolution capture of neuromuscular activity during real-world functional tasks. As emphasized in recent research, such integration enables a deeper understanding of subtle dynamics of movement and posture, which are critical to optimizing physical activity and designing inclusive mobility solutions. This study contributes to this growing field by applying sEMG to assess human–device interaction with a manually powered mobility platform.

## 2. Materials and Methods

A laboratory-based experimental protocol was conducted to evaluate upper limb muscle activity in women aged 50+ during simulated use of the Torqway personal mobility device. sEMG data were recorded across two movement phases: active and passive. The selection of muscles and measurement parameters was based on their functional involvement in operating the device.

### 2.1. Study Design and Participants

The research participants consisted of eleven women aged 50 and above, following the project’s assumptions. Participant recruitment was carried out among female residents of the Mazovian Voivodeship through announcements distributed via organizations promoting physical activity. Women aged 50 and above were eligible for inclusion. The inclusion criteria were as follows: overall good health, no medical contraindications to moderate physical activity, and the ability to move independently. Exclusion criteria included neurological or cardiac conditions, balance disorders, prior upper limb injuries, or implanted electronic devices (e.g., pacemakers). The sample size (*n* = 11) was determined based on the pilot nature of the study and the availability of participants meeting the inclusion criteria. Due to the exploratory character of the project and organizational constraints, no formal power analysis was conducted; however, the adopted sample size was sufficient to perform preliminary analyses of muscle activity differences between the examined movement phases. The characteristics of the research participants are presented in Table 1. The research was preceded by obtaining approval from the Ethics Committee: Komisja Etyki i Bioetyki Uniwersytetu Kardynała Stefana Wyszyńskiego w Warszawie. Additionally, each study participant was examined by a physician and signed a consent form to participate in the experiment.

The average age was 54.36 years. Due to the relatively small size of the research participants, participants were selected to ensure homogeneity in terms of physical fitness and build.

### 2.2. Test Environment and Equipment

The experimental research was carried out at the Faculty of Transport of the Warsaw University of Technology. The following section will present the results concerning the electromyographic analysis of the activity of selected muscles while the subject was riding on a Torqway. The analysis was based on parameters characterizing the sEMG signal, determined in the time domain (RMS amplitude) and in the frequency domain (mean power frequency, MPF). Another results of the analysis of body movement kinematics were published in [21].

#### Description of the Torqway Device

The Torqway is a manually powered personal mobility device designed to promote upper limb activity. It stands approximately 150 cm tall and weighs around 25 kg. The vehicle is propelled through a lever mechanism, requiring the user to push and pull the handlebars in a coordinated manner. Each lever movement contributes directly to the rotation of the drive axle, ensuring that there is no idle motion. Turning is achieved by shifting foot pressure: pressing the left platform turns the vehicle left, and pressing the right platform turns it right. The device reaches speeds of up to 12 km/h and can be operated in a standing position.

### 2.3. Surface Electromyography (sEMG) Measurement

**Figure 2:**
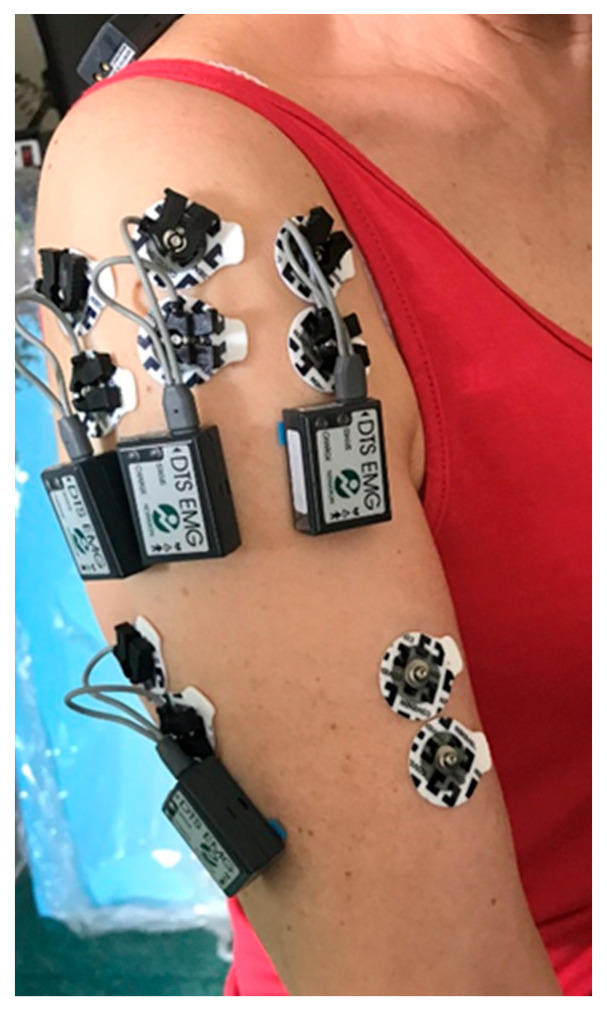
Placement of measurement sensors on the upper limb.

The sEMG signal was recorded using Noraxon MR software version 3.10.64 [22]. Bipolar surface electrodes were placed according to SENIAM guidelines on the following muscles of the dominant side: triceps brachii, anterior and posterior deltoid, and trapezius. The inter-electrode distance was set at 20 mm. Data were sampled at a frequency of 2000 Hz, band-pass filtered (20–450 Hz), and rectified. RMS (Root Mean Square) and MPF (mean power frequency) indicators were extracted for further analysis to quantitatively assess muscle activity and fatigue [23]. The electrode module, shown in Figure 2, consists of several key components used for collecting electromyographic (sEMG) data.

### 2.4. Estimation of Time and Frequency-Domain sEMG Signal Parameters

The first step of the analysis was to determine the Root Mean Square (RMS) amplitude for the signal recorded at rest (RMSrel) and during muscle activation under conditions similar to maximum load (RMSref). Then, based on the signal recorded during Torqway device riding, RMS amplitude and mean power frequency (MPF) were determined. To calculate RMS and MPF parameters, the sEMG signal was divided into windows of 1 s length. The sampling frequency of the sEMG signal was 1500 Hz; thus, a 1 s time window contained 1500 consecutive signal values. From each window, one RMS parameter value and one MPF parameter value were calculated. To normalize the amplitude, for each of the muscles studied, RMS values calculated from the sEMG signal recorded during Torqway riding (RMStg) were divided by the RMS values calculated from the reference signal recorded during muscle activation under conditions similar to maximum load (RMSref), and then multiplied by 100. The normalization of the sEMG signal amplitude is represented by Equation (1).

(1) $$RMS=\frac{RMStg}{RMSref}\cdot 100\;[\%\;ref]$$

Normalization of the RMS parameter values aimed to minimize the influence of individual factors (such as electrical resistance of the skin–electrode connection or signal attenuation by subcutaneous layers) on the amplitude of the sEMG signal.

Electromyographic signal analysis was performed using MATLAB ver. 2019. For each muscle and for both movement phases (active and passive), a set of time- and frequency-domain indicators was extracted.

In the frequency domain, a fast Fourier transform (FFT) with a segment length of 1024 points and Hamming windowing was applied. Two indicators were extracted: mean power frequency (MPF) and median frequency (MDF). MPF was calculated as follows (Equation (2)):

(2) $$MPF=\frac{\int_{0}^{\infty }fS\left(f\right)df}{\int_{0}^{\infty }S\left(f\right)df}$$

In summary, the integration of time- and frequency-domain analysis enabled a comprehensive evaluation of muscle function during the simulated propulsion task. The RMS parameter provided a quantitative measure of muscle activation intensity, while MPF and MDF allowed for the identification of spectral changes potentially associated with fatigue. This dual approach facilitated a more precise assessment of both the immediate neuromuscular response and time-related adaptations, supporting the overall aim of the study to characterize upper limb muscle performance during the use of a manually powered mobility device.

### 2.5. Statistical Analysis

To confirm the results regarding muscle activity during the use of the Torqway device, a statistical analysis of the data collected during the study was conducted. The focus was on comparing the values of RMS and MPF parameters in different phases of muscle activity (active and passive) and assessing the variability of these parameters over time. The statistical analysis aimed to verify hypotheses regarding significant differences in muscle activity between phases and potential signs of muscular fatigue. The following statistical tests were applied in the analysis: dependent samples *t*-test—to compare RMS and MPF between the active and passive phases; repeated measures ANOVA—to assess the variability of parameters over time; and Pearson correlation—to analyze the relationship between variables, such as BMI and muscular fatigue.

Normalization of RMS values was carried out according to Formula (1). Calculation of the average MPF power frequency was performed according to Formula (2).

The *t*-test for dependent samples was used to analyze differences between the active and passive phases. Its mathematical form is as follows:

$$t=\frac{\bar{d}}{\frac{s_{d}}{\sqrt{n}}}$$

where

$\bar{d}$—mean difference between pairs;
*s_d_*—standard deviation of the differences;
*n*—number of pairs of observations.

An analysis of variance was used to assess differences between measurements at different times:

$$F=\frac{{MS}_{within}}{\begin{array}{c} {MS}_{between} \end{array}}$$

where

${MS}_{within}$—mean square within groups;
${MS}_{between}$—mean square between groups.

## 3. Experimental Studies

### 3.1. Muscles Tested

**Figure 3:**
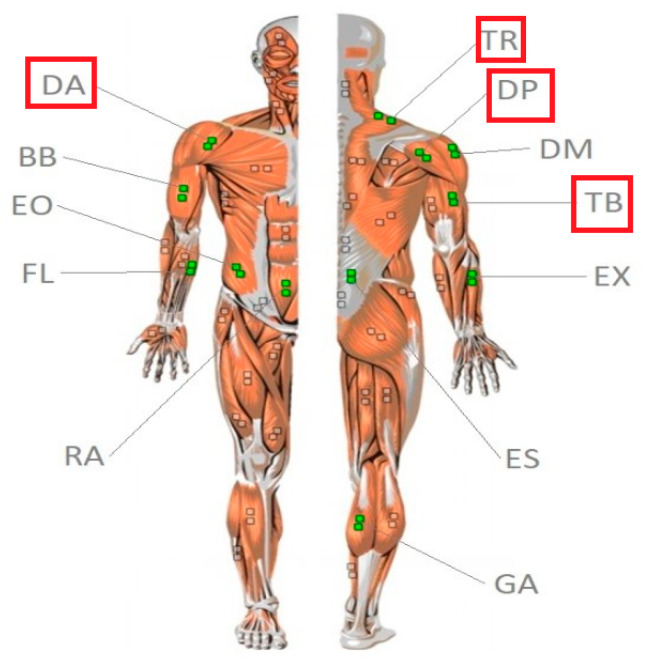
Muscle abbreviations. Source: Own elaboration based on https://hermanwallace.com/download/The_ABC_of_EMG_by_Peter_Konrad.pdf. (accessed on 25 May 2025). Where: Triceps brachii (TB), Anterior deltoid (DA), Posterior deltoid (DP), Upper trapezius (TR).

In the experimental procedure, surface sEMG signals were collected from twelve muscles. However, for the purpose of this article, we focused the analysis and interpretation of the results on four upper-body muscles—triceps brachii (TB), anterior deltoid (DA), posterior deltoid (DP), and upper trapezius (TR). These muscles were selected due to their consistent signal quality and functional involvement during the operation of the Torqway device. The remaining muscles, although recorded, showed less significant or inconsistent activation patterns under the tested conditions and will be considered in future publications. It was assumed that riding the Torqway device occurs symmetrically; therefore, only the muscles on the right side of the body were included in the studies. The selected muscles for activity assessment represent different muscle groups of the upper and lower body, which are involved in maintaining posture, controlling limb movements, and stabilizing the body during dynamic activities such as riding the Torqway. This approach was also guided by expert interviews with rehabilitation specialists who worked on the project. The muscles examined are presented in Figure 3.

### 3.2. Experimental Procedure

After placing the sensors, the sEMG signal was recorded for each of the examined muscles both at rest (referred to as the “relaxed” signal) and during muscle activation under conditions similar to maximum load (referred to as the “reference” signal). To allow interindividual comparison and reduce the influence of skin impedance and subcutaneous tissue on the EMG signal amplitude, normalization was performed with respect to the maximum voluntary contraction (MVC) for each muscle. MVC tests were conducted under standardized, isometric conditions for each muscle group separately. Participants were verbally encouraged to exert maximal effort for each test, and each contraction lasted 3–5 s, with 1–2 min of rest between attempts to prevent fatigue. In the article, the standardization and subsequent normalization to MVC were described only for four muscles: triceps brachii (TB), anterior deltoid (DA), posterior deltoid (DP), and upper trapezius (TR).

For consistency and reproducibility across participants, the following joint positions and resistance setups were used during MVC testing:

- Triceps brachii (TB):
  Elbow flexed at 90°, shoulder in neutral position. Participants performed an isometric elbow extension against manual resistance applied at the distal forearm.
- Anterior deltoid (DA):
  Shoulder flexed to 90°, elbow extended, palm facing down. The participant resisted a downward force applied just above the wrist to produce isometric shoulder flexion.
- Posterior deltoid (DP):
  Shoulder abducted to 90° in the horizontal plane (reverse fly position), elbow flexed at 90°. The participant exerted isometric horizontal abduction against resistance applied at the lateral elbow.
- Upper trapezius (TR):
  Shoulder in neutral position, participant elevated (shrugged) the shoulder isometrically against manual downward pressure applied to the acromion process.

To ensure data quality, three MVC attempts were performed per muscle. The trial with the highest RMS amplitude was used as the reference value for normalization. The EMG signals recorded during Torqway riding were then expressed as a percentage of this maximum value (%MVC), enabling relative comparisons across participants and conditions.

Subsequently, the sEMG signal was recorded during simulated Torqway riding (participants performed movements similar to natural Torqway riding, but the device was mounted on a stationary research stand). During the experimental trials, movement speed was controlled using a metronome set to 50 repetitions per minute to ensure consistency across participants. Force output was standardized by calibrating the resistance applied to the Torqway levers based on pre-test measurements. The pace of movement was maintained throughout the 10 min test sessions by providing verbal reminders to participants, ensuring uniformity in the experimental conditions. The 10 min duration of the simulated Torqway riding session was selected to provide a balance between ecological validity, participant comfort, and the ability to detect neuromuscular changes such as muscle fatigue. Previous studies on dynamic upper-body exercise, such as arm ergometry or Nordic walking […], have demonstrated that moderate-duration efforts of 8–12 min are sufficient to induce observable changes in EMG parameters, particularly in older adults.

Additionally, a longer duration could risk excessive fatigue or disengagement in participants unaccustomed to prolonged physical activity. The 10 min timeframe enabled participants to perform the repetitive motion at a controlled cadence (50 cycles/min) for a total of approximately 500 cycles, which was deemed sufficient to elicit physiological adaptation or early fatigue indicators (e.g., increased RMS or decreased MPF).

While not intended to induce maximal exhaustion, the protocol duration was designed to simulate a typical short-distance usage scenario in a real-world context (e.g., travel through a park or urban zone), ensuring both functional relevance and practical feasibility for future interventions.

**Figure 4:**
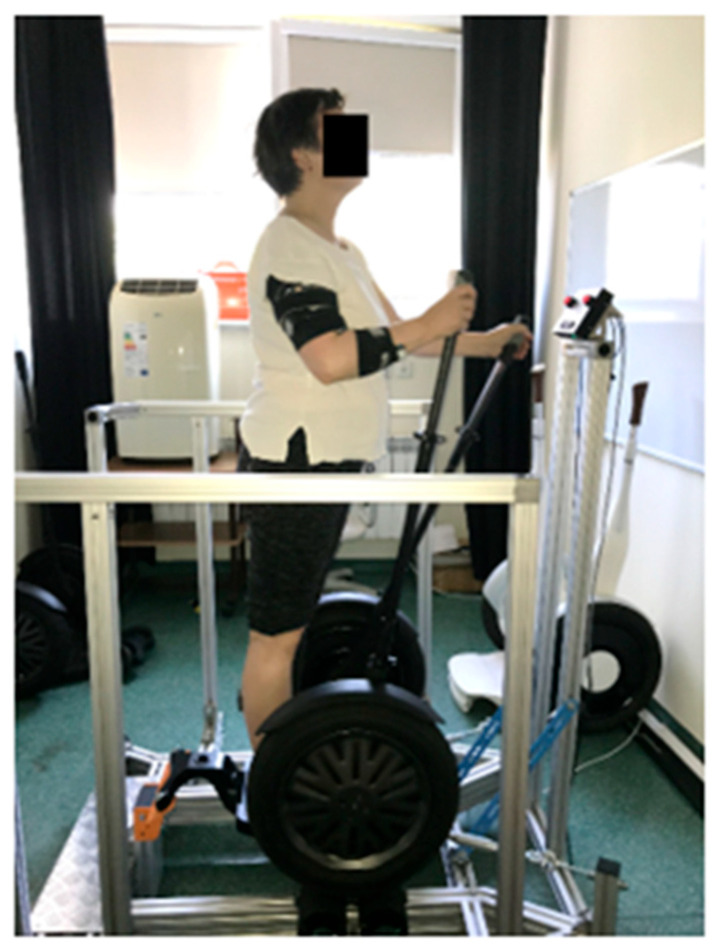
Participants on the stationary experimental setup.

The procedure for conducting experimental studies for representative participants is illustrated in Figure 4. All 11 participants completed the full 10 min test without interruptions or procedural modifications [24,25,26].

In the context of the study evaluating muscle activity during the use of the Torqway device, understanding the active and passive phases of muscle engagement is crucial for interpreting the results accurately. These phases are distinguished based on the nature of muscle activation during the cycling motions performed on the Torqway. The active phase was defined as periods in which the Root Mean Square (RMS) value of the triceps brachii exceeded 25% of the participant-specific maximum RMS (RMSmax), maintained for a minimum duration of 300 milliseconds. These thresholds were used to identify segments of voluntary muscular effort corresponding to propulsion movements while using the Torqway. The passive phase was defined as periods during which the RMS signal remained below the 25% RMSmax threshold, indicating minimal or no voluntary muscle activation. This typically included short recovery intervals between propulsion cycles. Phase classification was applied to all recorded muscle groups and was verified using synchronized video recordings to ensure consistency between signal-based segmentation and observed lever movements.

A marked increase in sEMG signal amplitude indicates an active phase, while a drop in amplitude indicates a transition to the passive phase.

By employing these methods, researchers can reliably categorize the muscle activity recorded during the experiment, providing insights into the muscle engagement patterns across different phases of Torqway usage. Understanding the active and passive phases is fundamental to the study’s objectives. It allows researchers to analyze how muscle activation varies with task demands, thereby providing insights into the biomechanics of using the Torqway device. This information can inform future improvements in design, optimize training protocols, and enhance the understanding of muscle dynamics in the context of novel transportation devices.

In electromyographic testing, it is necessary to determine a reference signal against which the values of signals recorded in specific tests are compared. Such a reference signal is the signal from the maximum muscle tension test. In a procedure in which the reference value is the maximum level of activation during contractions, the sEMG signal recorded during voluntary and spontaneous maximal contraction, expressing maximum muscle tension under isometric conditions, was used. The best results were obtained during the execution of isolated movement in a single joint, with static resting in the intermediate position of the range of motion.

## 4. Results

Following the analysis of the sEMG signal for each examined muscle and participant, 600 values of the RMS parameter and MPF parameter were obtained (each subsequent second of Torqway riding corresponds to one parameter value). The RMS and MPF parameter values derived from the sEMG signal underwent second-degree polynomial approximation, resulting in a trend line depicting parameter changes over the entire measurement duration. The analysis was performed using Noraxon’s MR3.14 software.

### 4.1. Analysis of Time-Dependent Muscle Activation

**Figure 5:**
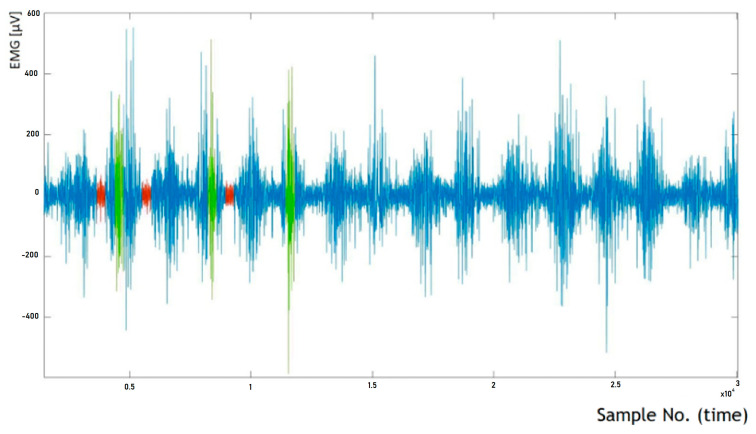
The method of determining the initial and final RMS amplitude values based on the raw sEMG signal (the first 20 s of recording are shown in the figure).

Torqway riding involves cyclic movements, distinguishable into “active” and “passive” phases of muscle activity. Another direction of sEMG signal analysis was to determine the initial and final RMS amplitude values based on the raw sEMG signal. For this purpose, characteristic segments of the signal from the first and last few tens of seconds of Torqway riding were selected (as close to the beginning and end of the measurement as possible, usually from the first and last twenty seconds of measurement). The method of determining initial and final RMS amplitude values based on the raw sEMG signal is illustrated in Figure 5.

Riding on the Torqway involves cyclic movements, where an “active” and “passive” phase of muscle work can be distinguished (as depicted in Figure 5; the active phase corresponds to segments with higher signal amplitude, while the passive phase corresponds to segments with lower amplitude). From both the initial and final segments of the signal, three segments corresponding to the active phase (marked in green) and three segments corresponding to the passive phase (marked in red) were selected. The length of each selected segment is 500 samples, which corresponds to a time interval of 1/3 of a second.

For each selected segment, the RMS amplitude was determined (which was also normalized). Subsequently, the RMS values from the three selected segments were averaged separately for the active and passive phases, and separately for the initial and final segments. As a result, one RMS parameter value was obtained for each of the following:

- The active phase from the initial seconds of riding (referred to as BH);
- The passive phase from the initial seconds of riding (BL);
- The active phase from the final seconds of riding (EH);
- The passive phase from the final seconds of riding (EL).

The determined initial and final RMS amplitude values based on the raw sEMG signal, corresponding to segments BH, BL, EH, and EL, enable both the assessment of changes in the RMS signal amplitude during the test duration and the determination of muscle tension levels during the active phase compared to the passive phase during Torqway riding, which can serve as a measure of muscle activation degree.

Below are selected results related to the following muscles: TB, DA, DP, and TR, which showed increased activity during driving.

It is worth emphasizing that if the muscle were fatigued, one would expect a simultaneous increase in the RMS amplitude of the sEMG signal and a decrease in the average MPF frequency. The following figures (from Figure 6, Figure 7, Figure 8, Figure 9, Figure 10, Figure 11, Figure 12, Figure 13, Figure 14, Figure 15, Figure 16 and Figure 17) display the results of the analyses for selected muscles.

For the triceps brachii muscle (TB), in all participants except for K2 and K4, there are noticeably higher values during the active phase of movement (BH and EH) compared to the passive phase (BL and EL). However, it should be noted that in participants K2 and K4, differences between the active and passive phases are also present, although not as pronounced as in the other participants.

**Figure 7:**
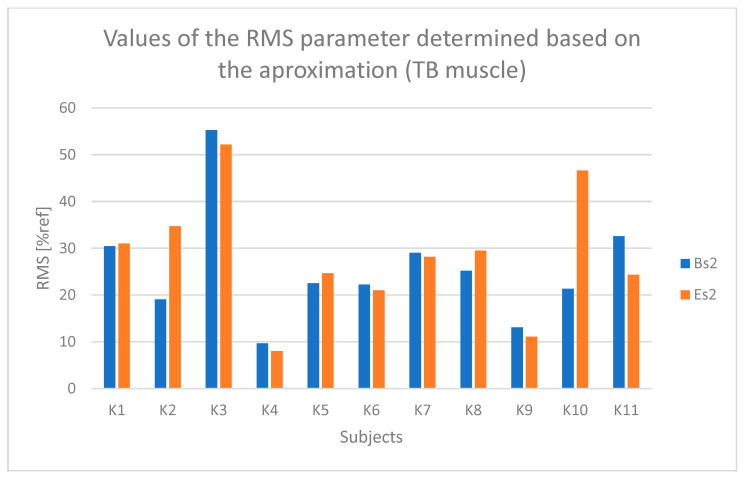
Values of the RMS parameter determined based on the approximation of the course from the triceps brachii muscle (TB) for all participants (Bs2—initial parameter value, Es2—final parameter value).

**Figure 8:**
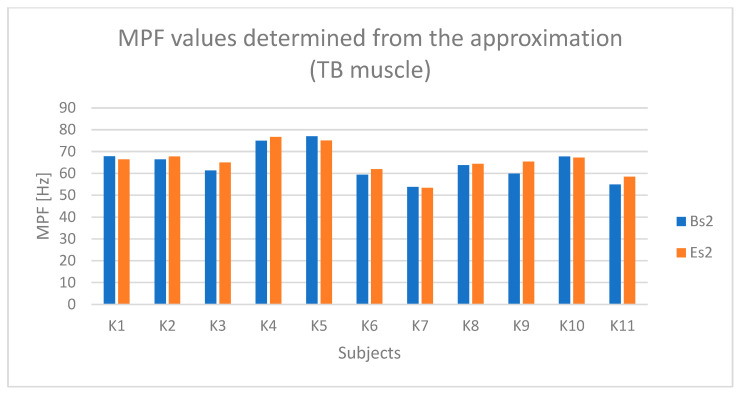
Values of the MPF parameter determined based on the approximation of the course from the triceps brachii muscle (TB) for all participants (Bs2—initial parameter value, Es2—final parameter value).

Based on Figure 7 and Figure 8, a slight trend indicating fatigue of the triceps brachii muscle can be observed in the case of participants K1, K5, and K10.

**Figure 9:**
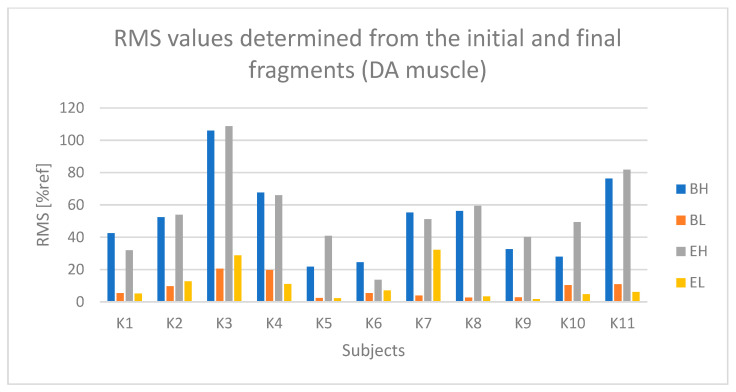
Values of the RMS parameter determined based on the initial and final segments of the raw sEMG signal from the anterior deltoid muscle (DA) for all participants (BH—active phase from the first seconds of the ride, BL—passive phase from the first seconds of the ride, EH—active phase from the last seconds of the ride, EL—passive phase from the last seconds of the ride).

In the case (Figure 9) of the anterior deltoid muscle (DA), higher values are observed during the active phase (BH and EH) compared to the passive phase (BL and EL). During the active phase, the amplitude for most participants ranged from about 40% to 80% of the reference value (% ref).

**Figure 10:**
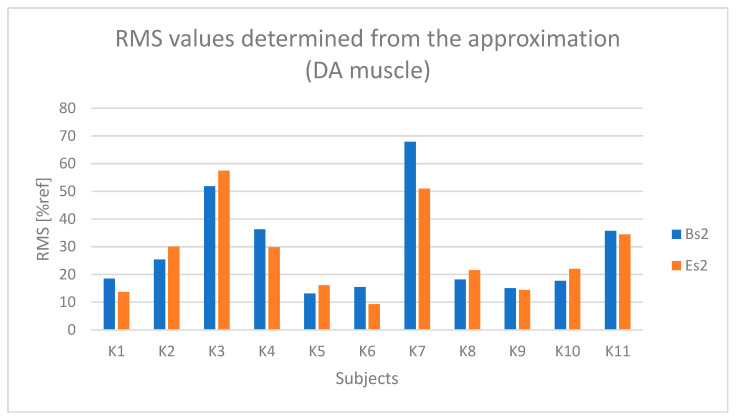
Values of the RMS parameter determined based on the approximation of the curve from the anterior deltoid muscle (DA) for all participants (Bs2—initial value of the parameter, Es2—final value of the parameter).

**Figure 11:**
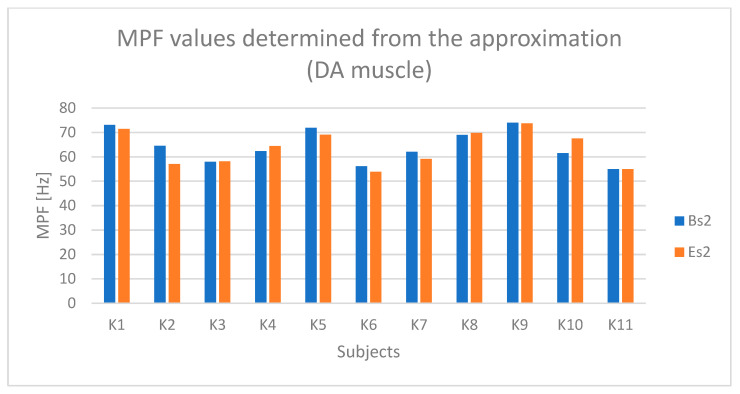
Values of the MPF parameter determined based on the approximation of the curve from the anterior deltoid muscle (DA) for all participants (Bs2—initial value of the parameter, Es2—final value of the parameter).

Based on Figure 10 and Figure 11, a relationship indicating fatigue of the anterior deltoid muscle (DA) can be observed in the case of participants K2 and K5.

**Figure 12:**
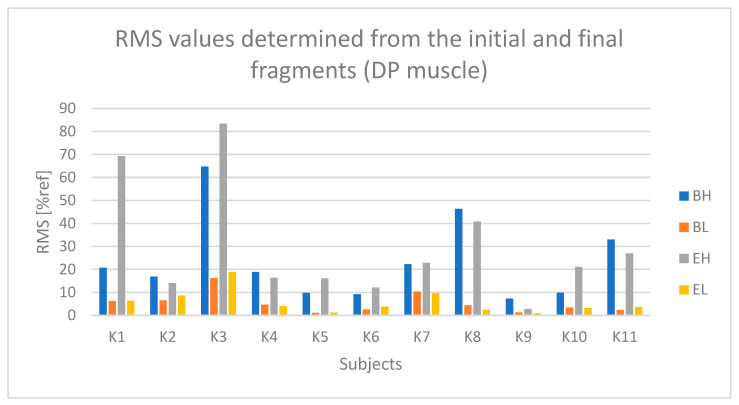
RMS parameter values determined from the initial and final fragments of the raw sEMG signal from the DP (posterior deltoid) muscle for all subjects (BH—active phase from the first seconds of driving, BL—passive phase from the first seconds of driving, EH—active phase from the last seconds of driving, EL—passive phase from the last seconds of driving).

The results concerning the posterior deltoid muscle (DP), similar to the DM muscle, indicate lower muscle activation (% ref) compared to the forearm and arm muscles (Figure 12). However, there is still a noticeable difference between the active (BH and EH) and passive (BL and EL) phases.

**Figure 13:**
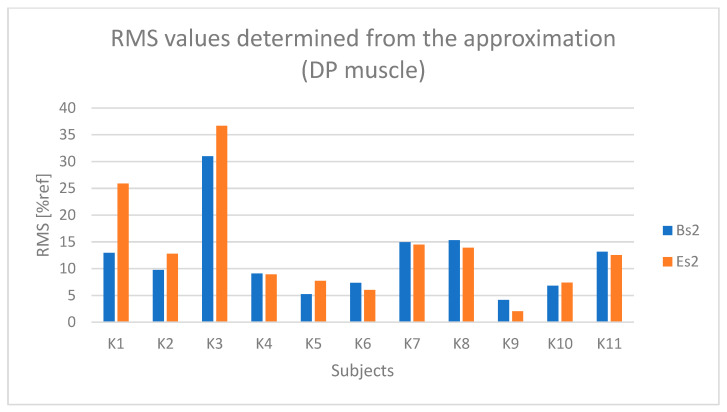
RMS parameter values determined based on the approximation of the DP (posterior deltoid) muscle signal for all examined subjects (Bs2—initial parameter value, Es2—final parameter value).

**Figure 14:**
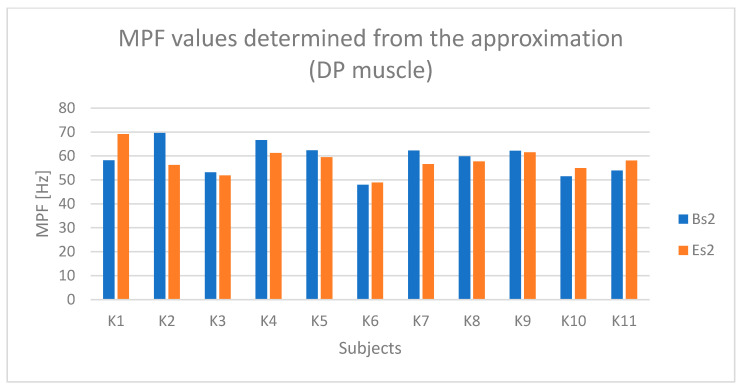
Values of the MPF parameter determined based on the approximation of the DP (posterior deltoid) muscle signal for all examined subjects (Bs2—initial parameter value, Es2—final parameter value).

Based on Figure 13 and Figure 14, a relationship indicating fatigue of the DP muscle can be observed for subjects K2, K3, and K5.

**Figure 15:**
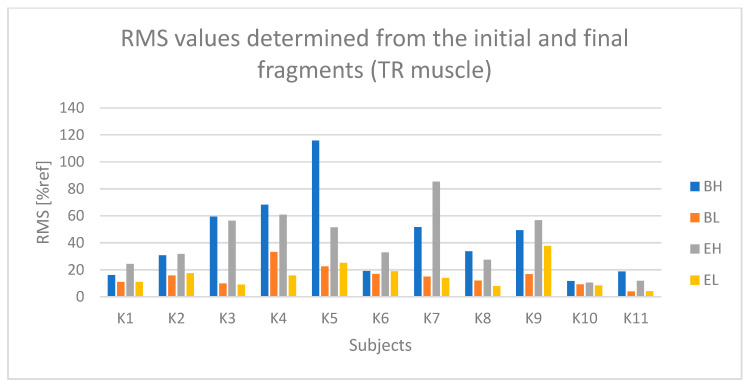
Values of the RMS parameter determined based on the initial and final segments of the raw sEMG signal from the TR (upper trapezius) muscle for all examined subjects (BH—active phase from the first seconds of riding, BL—passive phase from the first seconds of riding, EH—active phase from the last seconds of riding, EL—passive phase from the last seconds of riding).

In the case of the TR (upper trapezius) muscle (Figure 15), higher values were observed during the active phase (BH and EH) compared to the passive phase (BL and EL) for most examined subjects, although these differences were not as pronounced as in the previously analyzed muscles. During the active phase, the amplitude for most subjects ranged from 30% to 60% of the reference value (% ref).

**Figure 16:**
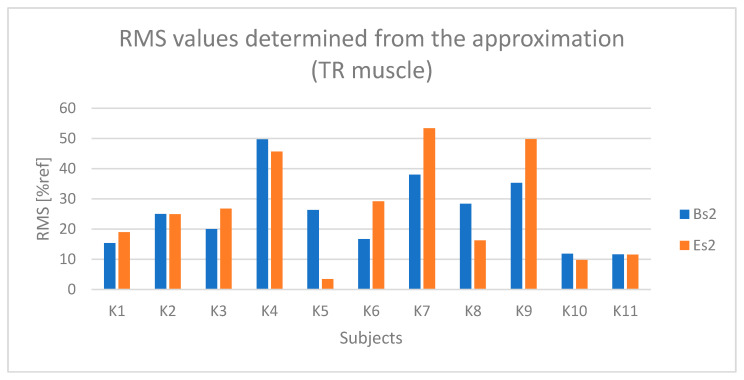
Values of the RMS parameter determined based on the approximation of the TR (upper trapezius) muscle signal for all examined subjects (Bs2—initial value of the parameter, Es2—final value of the parameter).

**Figure 17:**
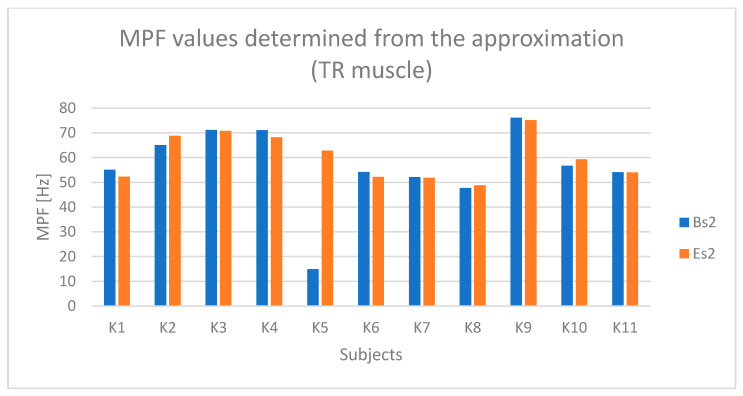
Values of the MPF parameter determined based on the approximation of the TR (upper trapezius) muscle signal for all examined subjects (Bs2—initial value of the parameter, Es2—final value of the parameter).

Based on Figure 16 and Figure 17, a relationship indicating the occurrence of fatigue in the TR muscle can be observed in the case of subjects K1, K3, K6, and K9.

Based on the obtained results, it can be concluded that for all analyzed muscles, significantly higher values occur during the active phase compared to the passive phase. This indicates a considerable activation of muscles during Torqway riding.

The analysis regarding the occurrence of muscle fatigue did not provide unequivocal results. A summary of cases where changes in sEMG signal parameters indicating muscle fatigue (simultaneous increase in RMS amplitude and decrease in mean MPF) were observed for the analyzed muscles is presented in Table 2. Studies confirming the relationship between muscle fatigue and changes in sEMG signal parameters, such as an increase in RMS amplitude and a decrease in mean frequency (MPF), have appeared in the literature for many years. References can be made, for example, to Sung, Lee [27], and Chowdhury et al. [28].

The relationship indicating the occurrence of muscle fatigue is most often observed in the case of individual K5. However, when discussing the involvement of muscles (DA, TB, TR) in the context of Torqway riding, we emphasize that while these muscles were indeed activated, their levels of engagement varied. Specifically, although the DP muscle showed lower activation compared to the primary movers, it still contributed to the overall activity during both the active and passive phases. This variability underscores the different roles that various muscle groups play during the exercise, where some muscles serve primarily for stabilization and support, while others are more engaged in generating movement. However, Table 3 presents the activation levels of analyzed muscles in the studied project at the Faculty of Transport, only those discussed in the article. It shows the percentage values of the RMS parameter (% ref), determined based on the initial segments of the raw sEMG signal for both the active and passive phases. The results are presented in the BH/BL configuration (BH—active phase from the first seconds of riding, BL—passive phase from the first seconds of riding) to better illustrate the examined issue.

### 4.2. Statistical Results

A statistical analysis was conducted to compare the normalized values of RMS and MPF parameters in the active and passive phases for selected upper limb muscles (Table 4). A paired *t*-test was applied to evaluate the significance of differences between the phases, while repeated measures ANOVA was used to assess the variability of parameters over time. The table below presents the mean ± standard deviation values for each phase, as well as the results of the statistical tests for the following muscles: triceps brachii (TB), anterior deltoid (DA), trapezius (TR), and posterior deltoid (DP). These results indicate significantly higher muscle activity during the active phase and demonstrate signs of muscle fatigue over the duration of the test.

The statistical comparison of the analyzed muscles shows a clear and consistent increase in RMS values during the active phase compared to the passive phase, which confirms higher neuromuscular involvement during physical effort using the Torqway device. Concurrently, the observed decrease in MPF values during the active phase reflects the onset of muscle fatigue, particularly in the triceps brachii and trapezius muscles. The statistically significant differences (*p* < 0.005 in all cases) support the hypothesis that prolonged use of Torqway induces measurable fatigue and can effectively engage upper limb muscles. These findings highlight the potential of Torqway as a tool for active mobility, inclusive physical activity, and even therapeutic training in rehabilitation or senior recreation settings.

## 5. Discussion

The paper discusses the results of muscle activation during Torqway riding, focusing on four upper limb muscles—the anterior deltoid (DA), posterior deltoid (PD), triceps brachii (TB), and trapezius (TR). These results indicate significant activation of these muscles in the active phase compared to the passive phase in individuals simulating Torqway riding.

The stationary tests were designed to minimize the risk of injury during actual riding due to insufficient training time before the tests. Additionally, a requirement set by the client was the characteristic of the study group, i.e., women aged 50 and older. The experimental studies were conducted at the Faculty of Transport of the Warsaw University of Technology using the Noraxon system.

Our study focused on evaluating muscle activity and fatigue during the use of the Torqway device, specifically by analyzing the Root Mean Square (RMS) and Median Power Frequency (MPF) of surface electromyographic signals (sEMG). The key findings from our research indicate that the RMS values, which reflect overall muscle activation levels, showed a significant increase during the exercise session. This suggests heightened muscle activity as participants engaged in repetitive movements with the Torqway device. Notably, the initial and final phases of the exercise cycle exhibited the most considerable differences in RMS values, reflecting changes in muscle engagement and potential fatigue over time.

The MPF values, which provide insights into the frequency characteristics of the sEMG signal, demonstrated a notable decrease towards the end of the exercise session. This decline is consistent with previous findings indicating that muscle fatigue is associated with a shift towards lower frequency components in the sEMG signal. The combined analysis of RMS and MPF provided a comprehensive view of muscle performance, revealing that as muscles fatigue, their activation patterns and frequency characteristics undergo significant changes.

However, sEMG alone cannot confirm fatigue definitively. Alternative explanations, such as motor unit synchronization or improved neuromuscular recruitment, are possible. No direct measures of torque or force were used, limiting interpretation.

Our study focused on specific segments of the exercise cycle, particularly the initial and final seconds, to capture the dynamic changes in muscle activity. This segmentation allowed for a detailed analysis of how muscle performance evolves during repetitive tasks. The methodological approach involved rigorous filtering of sEMG signals to remove motion artifacts, ensuring the accuracy and reliability of the data. By employing techniques such as low-pass filtering and visual inspection, we minimized the impact of noise and external disturbances on our measurements.

The insights gained can inform the design of training programs and rehabilitation protocols that aim to optimize muscle function and reduce the risk of injury. Future research could expand on these findings by exploring different exercise modalities, participant demographics, and incorporating advanced statistical analyses to further validate and extend our conclusions. While the study provides valuable insights into muscle activation patterns during the use of a personal mobility device, several limitations must be acknowledged. First, the sample size was relatively small (N = 11), and all participants were women aged 50 and above. This demographic limitation, although intentional, restricts the generalizability of the findings to broader populations, including younger users or men. Second, the tests were conducted in a stationary setting to minimize the risk of injury and maintain consistency, which may not fully capture the biomechanical dynamics of real-world riding scenarios.

Furthermore, although EMG data were collected from twelve muscles, the analysis focused on four upper-body muscles showing the highest and most consistent activation. This selection approach, while methodologically justified, may overlook synergistic or stabilizing roles of other muscles during riding.

Future studies should consider expanding the participant pool to include individuals of different ages, genders, and fitness levels to examine the variability in muscle engagement. Real-world testing conditions should also be incorporated to validate the results under dynamic environments. Additionally, combining sEMG with other sensor modalities, such as inertial measurement units (IMUs), pressure sensors, or motion tracking, could provide a more holistic biomechanical profile of user–device interaction. Longitudinal studies assessing changes in muscle performance, fatigue, or rehabilitation outcomes over time would also enhance understanding of the long-term impact of Torqway usage.

A hypothesis was formulated that it is possible to assess muscle activity in individuals using the Torqway personal mobility device through surface electromyography (sEMG). Torqway is a device that uniquely combines the most desirable features of electric vehicles, bicycles, and Nordic walking. Therefore, the research hypothesis that the personal mobility device activates the upper limb muscles discussed in the study can be confirmed. To confirm the hypothesis, the authors first reviewed studies utilizing the sEMG measurement technique. Since Torqway is an innovative device, these were pioneering studies, and there is no literature reference to research on this vehicle. However, there are studies on measuring upper limb muscle activity when using Nordic walking poles, which clearly indicate the activation of upper limb muscles. In studies by Zhang and Liu [29], the authors demonstrated that upper limb muscle activity significantly increased due to Nordic walking, especially the triceps brachii, similar to the studies on Torqway. In another study by Wang and Li [30], the effects of walking with and without Nordic walking poles on the activation of upper and lower limb muscles were compared. Their results indicate that using Nordic walking poles significantly increases the activation of both upper and lower limb muscles. Ribeiro, Tomeleri, and Schoenfeld [31] demonstrated that using similar types of devices, such as elliptical trainers, also significantly increases the activation of the upper and lower limb muscles. Romano, Sardella, and Zanetti [32] further confirmed the increased muscle activation during Nordic walking compared to traditional walking. Huang and Yu [33] specifically examined older adults and found significant muscle activation with Nordic walking, supporting the applicability of these findings to the Torqway. Therefore, the muscle activity observed during studies on the Torqway can be compared to these findings.

The presented literature studies expand our understanding of muscle activity during various forms of walking or moving on the Torqway and can be useful for conceptualizing the results of our study, which also focuses on analyzing muscle activation in response to specific stimuli associated with using recreational devices.

Furthermore, it is important to highlight the wide application of the sEMG method for such studies. It is most commonly used in athlete research or fields such as medicine and ergonomics, where it is necessary to demonstrate the activity of individual muscles or their fatigue. The results of studies conducted in this area are described in [1,2,6,9,10,16,21,22,34,35,36,37,38,39,40,41,42]. For example, scientific works [43,44] have shown that sEMG can be an effective tool for assessing movement patterns and muscle load under various physical conditions. In rehabilitation, sEMG is used to monitor therapy progress and design personalized exercise programs [45,46,47]. Studies indicate [48,49,50,51] that sEMG can also be useful for assessing muscle function, identifying muscle asymmetries, and conducting muscle training to restore full functionality after injuries or surgeries. In sports, sEMG is used to analyze movement techniques, assess muscle load, and monitor muscle fatigue [49,50]. In the field of ergonomic literature [21,52,53,54,55], studies suggest that sEMG can help identify risk factors for musculoskeletal injuries and assist in designing work environments that provide optimal ergonomic conditions.

This method also allows for the assessment of muscle function under various conditions, such as changes in body temperature, ambient temperature, or neuromuscular training. In the area of human–computer interaction, sEMG is used to control user interfaces using muscle signals, which can be an effective alternative to traditional control interfaces, especially for individuals with mobility limitations. Additionally, sEMG is used in diagnostics, treatment, sports training, and designing ergonomic technical objects, as described in [40,41,42].

In biomechanical studies, sEMG is often used to analyze muscle activity during various activities, such as walking, running, jumping, weightlifting, and riding different individual transportation means. From the perspective of the studies presented in the article, the results of these experiments are significant, focusing on muscle activation during riding on various individualized transportation means. This issue is addressed in works [1,2,18,31], and the presented research results have confirmed the effectiveness of this method. It is worth noting that the results described in this article pertain to pioneering studies of a patented personal mobility device developed under the Horizon 2020 program. The use of sEMG techniques by the authors to assess the suitability of the Torqway in terms of user fit and the potential benefits from activating selected muscle groups during riding represents an innovative research approach that undoubtedly requires further continuation. Further research could include the demographic diversity of participants, long-term studies on rehabilitation effectiveness, comparative analysis with other devices, and real-world usage conditions of the personal mobility device.

It is also worth mentioning the technical and measurement limitations of this method. Surface electromyography is susceptible to environmental interference, such as electromagnetic interference generated by electrical equipment, lighting, or other medical devices, which can affect measurement accuracy. Therefore, the authors thoroughly analyzed raw data and eliminated gross errors. Additionally, the sEMG signal must pass through the skin, subcutaneous tissue, and muscle tissue, which can lead to signal attenuation or distortion. Despite these limitations, the selected methods and measurement tools allowed for demonstrating muscle activity and justifying the use of the Torqway for both recreational and rehabilitation purposes.

These findings align with the broader goals of this Special Issue focused on sustainable transport and mobility. The Torqway, as a form of active, low-emission mobility, presents an innovative response to the challenges of overloaded conventional transportation systems, increasing emissions, and the need to adapt urban spaces for elderly users and individuals with limited mobility. Its compact design, zero emissions, and potential to engage upper limb musculature make it suitable for promoting active transport and recreational mobility in urban and touristic areas, supporting the achievement of Sustainable Development Goals related to transport, public health, and social inclusion.

From a technological standpoint, the study illustrates how electromyographic sensing and time–frequency data analytics can serve as practical tools for evaluating personalized transport solutions. Future work may extend this approach by integrating multimodal sensors (e.g., inertial measurement units, pressure sensors, motion tracking systems) to provide a more comprehensive biomechanical profile. Moreover, the ability to quantify muscle fatigue and activity in real-time supports potential applications in adaptive user interfaces, intelligent feedback systems, and rehabilitation robotics, which are central to the evolving landscape of human-centered transport innovation. Although our findings revealed increased RMS and decreased MPF values during the active phase of Torqway riding, which are typically associated with neuromuscular fatigue, it is important to consider alternative physiological explanations. These changes in sEMG parameters could also reflect improved motor unit recruitment strategies, increased voluntary drive, or motor learning adaptations over time, particularly as participants became more familiar with the movement pattern.

Furthermore, RMS and MPF changes can be influenced by factors unrelated to true fatigue. For example, increased motor unit synchronization, changes in skin impedance (e.g., due to perspiration), and variations in electrode placement or signal filtering may affect signal interpretation. While we minimized motion artifacts and maintained signal consistency, these confounding factors cannot be entirely excluded.

It is also important to note that no direct measures of muscular force, torque output, or perceived fatigue were collected during the experiment. Thus, although RMS and MPF are valuable indicators, they should not be viewed as definitive proof of muscular fatigue. Future studies should include complementary assessments—such as dynamometry, biochemical markers, or subjective fatigue ratings—to provide a more robust characterization of fatigue.

We acknowledge this limitation and recommend that future research incorporate multimodal indicators to improve the specificity and validity of sEMG-based fatigue assessment in personalized transport applications.

## 6. Conclusions

The study demonstrated that upper limb muscle activity is significantly higher during the active propulsion phase compared to the passive phase while using the Torqway. The observed decline in MPF supports the presence of early fatigue signs. These findings suggest the Torqway can be an effective tool for promoting physical activity and rehabilitation in older adults. Further field research is recommended, involving real-world testing of the device in urban and touristic settings, to better evaluate its impact on mobility, public health, and user quality of life. The findings highlight the relevance of wearable sEMG in advancing sensor-based assessment frameworks for sustainable and inclusive transport. The integration of such sensing technologies can pave the way for personalized ergonomic evaluation, real-time fatigue monitoring, and user-responsive mobility systems, supporting the broader vision of smart and health-oriented urban environments.

## 7. Limitations

One of the limitations of this study is the lack of direct measurement of subcutaneous fat thickness, which is known to affect the amplitude and quality of surface EMG signals. While participants’ BMI was recorded and used to ensure general physical homogeneity, variations in localized subcutaneous tissue could still have introduced interindividual differences in signal attenuation. Future studies should consider the inclusion of ultrasound or caliper-based fat measurements to improve the precision and interpretability of EMG data. Additionally, the study did not include direct measurements of muscle force output (e.g., torque, peak force) or subjective fatigue ratings (e.g., Borg scale), which limits the interpretation of fatigue based solely on sEMG parameters. Without complementary biomechanical or perceptual indicators, RMS and MPF changes cannot be unequivocally attributed to neuromuscular fatigue. Future research should integrate multidimensional fatigue assessment, including dynamometry, biochemical markers, and user-reported outcomes, to confirm physiological interpretations.

## Figures and Tables

**Figure 6:**
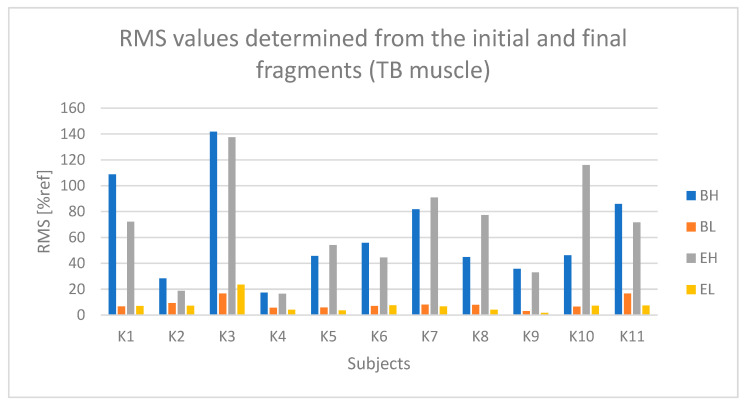
RMS parameter values determined based on the initial and final segments of raw sEMG signal from the triceps brachii muscle (TB) for all participants (BH—active phase from the first seconds of riding, BL—passive phase from the first seconds of riding, EH—active phase from the last seconds of riding, EL—passive phase from the last seconds of riding).

**Table 1:** Characteristics of the research participants.

Tested Person	Age	Height (cm)	Weight (kg)	BMI
k1	54	165	72	26.45
k2	54	164	79	29.37
k3	52	153	62	26.49
k4	59	162	69	26.29
k5	62	165	63	23.14
k6	50	167	90	32.27
k7	57	166	69	25
k8	50	170	57	19.7
k9	52	164	57	21.2
k10	58	161,5	62	23.92
k11	50	170	72	24.91

**Table 2:** Summary of cases where changes in sEMG signal parameters indicating muscle fatigue were observed during Torqway riding.

Muscle/ Person	TB	DA	DP	TR
K1	x			x
K2		x	x	
K3			x	x
K4				
K5	x	x	x	
K6				x
K7				
K8				
K9				x
K10	x			
K11				

**Table 3:** A summary presenting the activation levels of the analyzed muscles—percentage values of the RMS parameter (% ref), determined based on the initial segments of the raw sEMG signal for the active and passive phases of movement; the results are presented in the BH/BL configuration (BH—active phase from the first seconds of riding, BL—passive phase from the first seconds of riding).

Muscle/ Person	TB	DA	DP	TR
K1	109/ 7	42/ 5	21/ 6	16/ 11
K2	28/ 9	52/ 10	17/ 6	31/ 16
K3	142/ 17	106/ 21	65/ 16	59/ 10
K4	17/ 6	68/ 20	19/ 5	68/ 33
K5	46/ 6	22/ 2	10/ 1	116/ 23
K6	56/ 7	25/ 6	9/ 3	19/ 17
K7	82/ 8	55/ 4	22/ 10	52/ 15
K8	45/ 8	56/ 3	46/ 4	34/ 12
K9	36/ 3	33/ 3	7/ 1	49/ 17
K10	46/ 6	28/ 10	10/ 3	12/ 9
K11	86/ 17	76/ 11	33/ 2	19/ 4

**Table 4:** Statistical analysis active and passive phases for selected upper limb muscles.

Muscle	RMS Active (M ± SD)	RMS Passive (M ± SD)	Test *t* (*p*-Value)	MPF Active (M ± SD)	MPF Passive (M ± SD)	ANOVA (F, *p*)
TB	109 ± 7	28 ± 9	t(10) = 5.62, *p* < 0.001	120 ± 8	95 ± 10	F(1,10) = 12.34, *p* = 0.003
DA	106 ± 21	52 ± 10	t(10) = 4.92, *p* < 0.005	115 ± 9	92 ± 12	F(1,10) = 9.87, *p* = 0.007
TR	116 ± 23	31 ± 16	t(10) = 6.21, *p* < 0.001	112 ± 7	90 ± 11	F(1,10) = 11.45, *p* = 0.004
DP	98 ± 15	40 ± 12	t(10) = 4.35, *p* < 0.005	110 ± 9	88 ± 13	F(1,10) = 10.12, *p* = 0.005

## Data Availability

The original contributions presented in the study are included in the article; further inquiries can be directed to the corresponding author. Data are available upon request.
